# Vasoactive-inotropic agents in pediatric patients undergoing cardiac surgery: A single-center retrospective study

**DOI:** 10.1097/MD.0000000000042333

**Published:** 2025-05-02

**Authors:** Yan-ting Sun, Li-hong Wang, Yun-tai Yao

**Affiliations:** a Department of Anesthesiology, Baoji high-tech Hospital, Shaanxi, China; b Department of Anesthesiology, Fuwai Hospital, National Center for Cardiovascular Diseases, Peking Union Medical College and Chinese Academy of Medical Sciences, Beijing, China; c Department of Anesthesiology, Chuiyangliu Hospital of Tsinghua University, Beijing, China.

**Keywords:** agents, cardiac surgery, inotropic, pediatric, vasoactive

## Abstract

This study aimed to retrospectively summarize the use of vasoactive-inotropic agents in pediatric patients undergoing cardiac surgery at Fuwai Hospital. A total of 401 patients who met the screening criteria were enrolled in this study between April and June 2021 at Fuwai Hospital. We retrospectively summarized the current practices for vasoactive-inotropic agent use across different ages, Risk Adjustment in Cardiac Surgery 1 (RACHS-1) categories, and among various anesthesiologists. Intraoperatively, milrinone was the most commonly used inotrope (327 patients, 81.6%), followed by dopamine (274, 68.3%), dobutamine (263, 65.4%), epinephrine (67, 16.7%), and isoprenaline (11, 2.7%). Vasopressin was mainly administered during the pediatric intensive care unit period, with the highest use rate on postoperative day (POD)-1 (16/401, 3.9%). Furthermore, a combination of dopamine, dobutamine, and milrinone was administered by 52.1% of anesthesiologists intraoperatively and by 30.2% of pediatric intensivists on POD 1. Milrinone, dopamine, and dobutamine were selected by most anesthesiologists (13/14, 92.9%), and their usage rates among different anesthesiologists ranged from 66.67% to 92.68%, 52.94% to 89.66%, and 46.18% to 86.21%, respectively. Moreover, their use in category 4 surgeries was significantly higher than in category 1 to 3 surgeries. Milrinone, dopamine, and dobutamine were the most commonly used vasoactive-inotropic agents, while the other agents represented the diversity of medications used during both the intra- and postoperative periods in pediatric cardiac surgery at Fuwai Hospital.

## 1. Introduction

Cardiac dysfunction in pediatric patients undergoing cardiac surgery is influenced by factors such as immature myocardial injury, cardiopulmonary bypass, systemic inflammation, myocardial ischemia, and myocardial edema, which can lead to hemodynamic instability during and after surgery.^[1]^ Therefore, the use of vasoactive-inotropic agents to stabilize hemodynamics is a critical aspect of perioperative management in pediatric cardiac surgery.^[2]^

However, despite their critical role, the selection and administration of these agents vary significantly across countries, medical centers, and practitioners.^[3]^ Few studies have examined the perioperative use of vasoactive-inotropic agents in children with congenital heart disease (CHD), and existing guidelines are often shaped by local traditions and individual clinical experience rather than by robust, evidence-based protocols.^[4,5]^ This lack of uniformity contributes to practice variability and highlights the urgent need for comprehensive research in this area.^[6]^

Fuwai Hospital, a leading cardiovascular center in China, performs over 10,000 cardiac surgeries annually, of which pediatric cardiac operations account for more than 50%.^[7]^ Despite its prominence, no studies have yet summarized the use of vasoactive-inotropic agents in pediatric cardiac surgery, particularly regarding how drug selection varies by surgery type, patient age, and anesthesiologist experience. This study aims to fill this gap by providing a descriptive analysis of the usage patterns of vasoactive-inotropic agents at Fuwai Hospital, offering data that may inform future practices at this and other institutions in pediatric cardiac surgery.

## 2. Materials and methods

### 2.1. Patients and data collection

**Figure 1.**
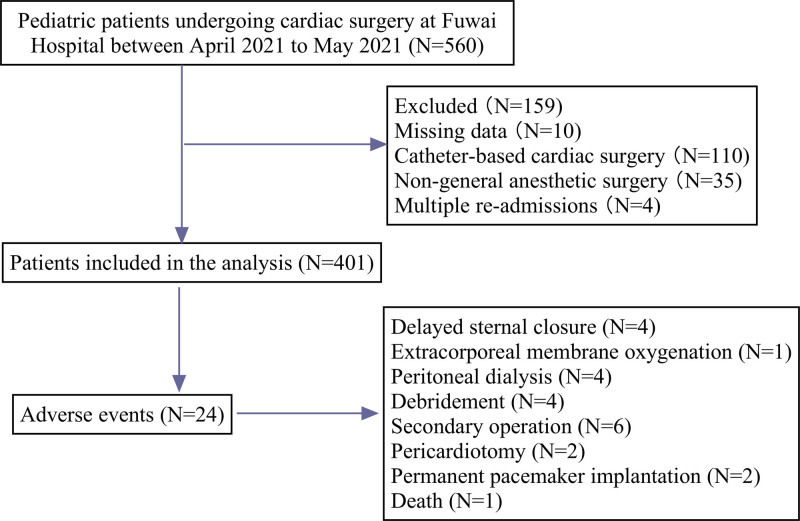
Patient inclusion.

This study is a secondary analysis of a previous study conducted by the authors.^[8]^ The research was performed at Fuwai Hospital, Chinese Academy of Medical Sciences, a single tertiary care center with dedicated pediatric cardiovascular operating rooms and a pediatric intensive care unit (PICU). The study was approved by the Ethical Committee of Fuwai Hospital (2014zlgc0759), and informed consent was waived due to its retrospective, observational nature. Patients aged ≤ 18 years who underwent congenital heart surgery with cardiopulmonary bypass (CPB) between April and June 2021 were enrolled. A total of 401 patients met the inclusion criteria, as shown in Figure 1. All procedures and protocols followed relevant guidelines and regulations.

Demographic, clinical, and short-term prognostic data were collected from the Anesthesia Information System and the Hospital Information System at Fuwai Hospital. Intraoperative clinical data included sex, age, weight, height at the time of surgery, primary procedure performed, aortic cross-clamping (ACC) duration, CPB duration, total operation time, urine output, bleeding volume, CPB priming volume, intravenous infusion, serum glucose and lactate measured before and after CPB, as well as adverse events. Data on the types of vasoactive-inotropic agents used intraoperatively (intra-op) and in the subsequent 2 hours (post-op-2h), 1 day (POD-1), and 2 days (POD-2) after arrival at the PICU were also collected. Adverse events were defined as any of the following occurrences or interventions: delayed sternal closure, extracorporeal membrane oxygenation, peritoneal dialysis, permanent pacemaker implantation, debridement, reoperation, pericardiotomy, or death.^[8]^

### 2.2. Vasoactive-inotropic drugs

At our center, vasoactive-inotropic agents are typically initiated by the attending anesthesiologist and cardiac surgeon in the operating room once rewarming begins. The dosage and choice of agents are then adjusted according to individual patient characteristics, clinical condition, central blood pressure, and overall physiological status. These agents are administered via continuous intravenous infusion using electronic infusion pumps. Upon arrival at the Pediatric Intensive Care Unit (PICU), adjustments to or discontinuation of vasoactive-inotropic agents, are made collaboratively by the cardiac surgeon and pediatric intensivist, based on the patient’s clinical status.^[8]^ The primary inotropes administered via continuous intravenous infusion include dopamine, dobutamine, milrinone, epinephrine, and isoproterenol. The main vasopressors are norepinephrine and vasopressin, while vasodilators such as nitroglycerin, sodium nitroprusside dihydrate (SNP), recombinant human brain natriuretic peptide, phentolamine, and treprostinil are also available as needed.

### 2.3. Endpoints

The objective of this study was to describe current practices regarding vasoactive-inotropic agent use during the intraoperative period, as well as at post-op-2h, POD-1, and POD-2, across different age groups, Risk Adjustment in Cardiac Surgery 1 (RACHS-1) categories, and among various anesthesiologists at Fuwai Hospital. Given the wide range of surgical procedures (more than 150) performed for CHDs, the use of the vasoactive-inotropic agents in different operations was categorized according to the RACHS-1 method (categories 1–6), as described by Cavalcanti PE.^[9]^ This method categorizes procedures based on complexity using surgical procedures rather than CHD diagnoses.

### 2.4. Statistical analysis

All data were collected and recorded in duplicate using Microsoft Excel. Figures were generated with GraphPad Prism (version 8.0). Statistical analyses were conducted using SPSS (version 22.0; SPSS Inc., Chicago). Results are presented as absolute frequencies, proportions (%), or medians with ranges. Descriptive statistics were used to analyze the data.

## 3. Results

### 3.1. Patients

A total of 401 patients who met the screening criteria were enrolled in this study between April and June 2021 (Fig. 1). The mean age was 30.12 ± 21.61 months, and more than half of the patients were female. The median duration of ACC was 40.3 minutes, with a spontaneous resuscitation rate of 91.8%. Pediatric cardiac procedures had an average duration of 163.1 ± 84.1 minutes, with 78.6% of surgeries performed as the first or second case of the day. The detailed baseline demographic and clinical characteristics are presented in Table 1.

**Table 1:** Intra-operative clinical data.

Baseline		Data (N = 401)
Sex	Female	214 (53.4)
	Male	187 (46.6)
Age, mo		30.12 ± 21.607
Weight, kg		12.06 ± 5.31
Height, cm		86.26 ± 19.97
ACC duration, min*		40.3(21.9, 66.6)
CPB duration, min*		64.0(45.0, 101.9)
Operation duration, min		163.1 ± 84.1
Spontaneous resuscitation	Yes	368 (91.8)
Intra-operative	Urine volume, mL	467.1 ± 199.7
	Bleeding volume, mL*	39.7(24.4, 64.4)
	CPB priming volume, mL	356.1 ± 125.5
	Iv. infusion, mL*	64.1 (48.9, 78.1)
POD-1	Total fluid out, mL	602.5 ± 463.3
	Total fluid in, mL	524.6 ± 334.6
POD-2	Total fluid out, mL	767.4 ± 399.0
	Total fluid in, mL	644.7 ± 343.9
Serum glucose, mmol/L	Pre-CPB	4.97 ± 1.01
	Post-CPB	7.27 ± 2.25
Serum lactate, mmol/L	Pre-CPB	0.97 ± 0.56
	Post-CPB	1.63 ± 0.98
MVD, h*		5.1 (5.0, 22.9)
LOS in the hospital, d*		10.2 (7.8, 14.2)

Data reported as a mean ± standard deviation, categorical data are expressed as n (%).

ACC = aortic cross-clamping, CPB = cardiopulmonary bypass, DSC = delayed sternal closure, ECMO = extracorporeal membrane oxygenation, LOS = length of stay, MVD = mechanical ventilation duration, PD = peritoneal dialysis, PPI = permanent pacemaker implantation.

* Continuous data with non-normal distribution are expressed using the median (25th, 75th percentile). CPB priming volume includes machine priming and additional crystals, colloids, and blood in the machine. Intravenous infusion includes all crystals, colloids, and blood entered from the peripheral and central veins throughout the procedure.

### 3.2. Intra- and post-operative administration of vasoactive-inotropic agents

All 401 patients received at least one inotrope or vasoactive agent.

**Figure 2.**
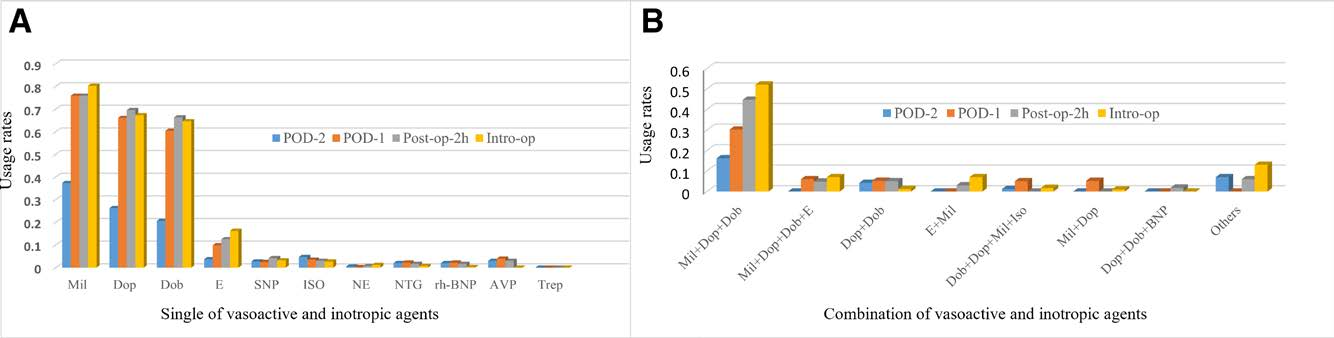
Single (A) and combination (B) of vasoactive and inotropic agents during intra-op and subsequent post-op-2h, POD-1 and POD-2. Dob = dobutamine, Dop = dopamine, E = epinephrine, Intra-op = Intra-operative, Iso = isoproterenol, Mil = milrinone, NE = norepinephrine, NTG = nitroglycerin, POD = post-operative day, Post-op = post-operative, rh-BNP = recombinant human brain natriuretic peptide, SNP = sodium nitroprusside dihydrate, Trep = treprostinil, Vaso = vasopressin.

Intraoperatively, milrinone was the most commonly used inotrope (327 patients, 81.6%), followed by dopamine (274, 68.3%), dobutamine (263, 65.4%), epinephrine (67, 16.7%), and isoprenaline (11, 2.7%). Norepinephrine was the most commonly used vasopressor (5/401, 1.4%), whereas the most frequently used vasodilator was SNP (13/401, 3.2%). During the postoperative period, the top three inotropes – milrinone, dopamine, and dobutamine – were consistently used at post-op-2h, POD-1, and POD-2, similar to intraoperative usage. Vasopressin was primarily used during the PICU period, with its highest usage rate on POD-1 (16/401, 4.0%). Additionally, the combination of dopamine, dobutamine, and milrinone was the most commonly used inotrope regimen, administered by 52.1% of anesthesiologists intraoperatively and by 30.2% of pediatric intensivists on POD-1 when a single agent was insufficient for inotropic support. Other combinations, including dopamine, dobutamine, milrinone, and epinephrine, were used by 7.5% of anesthesiologists intraoperatively and 5.8% of pediatric intensivists on POD-1. Other vasoactive drugs used in this study included nitroglycerin, SNP, recombinant human brain natriuretic peptide, and phentolamine (Fig. 2).

### 3.3. RACHS-1 categories and vasoactive-inotropic agents

The RACHS-1 distribution for pediatric surgical procedures was as follows: category 1 (17/401, 4.2%), category 2 (231/401, 57.6%), category 3 (143/401, 35.6%), category 4 (9/401, 2.2%), category 5 (0%), and category 6 (1/401, 0.2%). Milrinone, dopamine, and dobutamine were the most commonly used inotropes across all RACHS-1 categories, both intraoperatively and on POD-2. Their usage of these agents was significantly higher in category 4 compared with categories 1–3. SNP was the most frequently used vasodilator, but its usage rate in category 1 procedures dropped significantly on POD-2 (5.9%) compared with its rates intraoperatively (58.8%), at post-op-2h (58.8%), and on POD-1 (35.3%) (Table 2).

**Table 2:** Prevalence of vasoactive and inotropic drugs used in surgical procedures of different RACHS-1 categories.

RACHS-1	Dop	Dob	Mil	E	NE	AVP	Iso	NTG	SNP	rh-BNP	Trep
Intra-op											
Category 1	64.7%	64.7%	82.4%	11.8%			5.9%	5.9%	58.8%		
Category 2	68.4%	67.9%	84.4%	10.8%			1.7%	0.4%	0.4%		
Category 3	73.4%	67.1%	84.6%	23.8%			4.2%	0.7%	1.4%	0.7%	
Category 4	88.9%	88.9%	88.9%	88.9%							
Category 6	100%										
Post-op-2 h											
Category 1	11.8%	5.9%	17.6%				58.8%	58.8%	58.8%		
Category 2	71.0%	24.7%	58.8%	8.0%		3.0%	2.0%	1.0%	1.0%	1.0%	
Category 3	69.0%	30.0%	84.0%	19.0%	2.0%	2.0%	3.0%	3.0%	3.0%	2.0%	
Category 4	88.8%	66.6%	88.8%	66.6%		33.3%	22.2%		11.1%	11.1%	
Category 6		100%	100%								
POD-1											
Category 1	5.9%	5.9%	17.7%				5.9%	5.9%	35.3%		
Category 2	69.7%	64.9%	77.1%	6.9%		3.5%	1.7%	1.3%	0.4%	1.3%	
Category 3	69.2%	61.5%	83.9%	13.9%		4.9%	4.9%	3.5%	2.1%	3.5%	
Category 4	88.9%	88.9%	88.9%	44.5%		11.1%	22.2%			11.1%	
POD-2											
Category 1	5.9%	5.9%	5.9%						5.9%		
Category 2	24.7%	24.7%	33.8%	3.1%		2.2%	2.6%	0.9%	1.7%	1.7%	0.4%
Category 3	30.1%	23.1%	45.5%	4.2%	1.4%	2.8%	7.7%	4.2%	2.8%	2.1%	4.2%
Category 4	66.7%	66.7%	88.9%	33.3%		33.3%	22.2%		22.2%	11.1%	
Category 6		100%									

The RACHS‐1 category 1 of the pediatric surgical procedures was 4.2% (17/401), of which 16 required PDA ligation and 1 required OS ASD closure. The RACHS‐1 category 2 was 57.6% (231/401), of which 195 required VSD or OP ASD closure, 15 required TOF repair, 14 required TAPVC repair, 5 required AC repair and 2 required glenn procedure. The RACHS‐1 category 3 of the pediatric surgical procedures was 35.6% (143/401), of which 132 required tricuspid valve replacement or mitral valve replacement, 7 required S-PA shunt, 2 required ross procedure and 2 required PA banding. The RACHS-1 category 6 was 0.2% (1/401), of which required blolock-faussing procesure.

AVP = vasopressin, Dob = dobutamine, Dop = dopamine, E = epinephrine, Intra-op = Intra-operative, Iso = isoproterenol, Mil = milrinone, NE = norepinephrine, NTG = nitroglycerin, POD = post-operative day, Post-op = post-operative, rh-BNP = recombinant human brain natriuretic peptide, SNP = sodium nitroprusside dihydrate, Trep = treprostinil.

### 3.4. Ages and vasoactive-inotropic agents

The 401 patients recruited in this study included seven newborns (1.7%; age < 30 days), 106 infants (26.3%; age 30 days–1 year), 140 toddlers (35.0%; age 1–4 years), and 148 children (36.8%; age 4–18 years). The four most commonly used inotropes across all age groups were milrinone, dopamine, dobutamine, and epinephrine. In the intraoperative period, the usage rates of milrinone, dopamine, dobutamine, and epinephrine were 100.0%, 100.0%, 85.7%, and 71.4%, respectively, in newborns; 84.9%, 80.1%, 78.3%, and 23.6% in infants; 80.0%, 63.6%, 60.7%, and 17.9% in toddlers; and 85.8%, 66.9%, 63.5%, and 9.4%, respectively, in children. During the postoperative period, the most commonly used inotropes remained similar to those used intraoperatively. Their usage decreased significantly on POD-2, whereas vasopressin and SNP use increased, with rates of 42.9% and 14.3%, respectively, in newborns (Table 3).

**Table 3:** Prevalence of vasoactive and inotropic drugs used in pediatric patients at different ages.

Ages	Dop	Dob	Mil	E	NE	AVP	Iso	NTG	SNP	rh-BNP	Trep
Intra-op											
Newborns	100%	85.7%	100%	71.4%							
Infants	80.1%	78.3%	84.9%	23.6%	0.9%		5.7%		3.8%	0.9%	
Toddlers	63.6%	60.7%	80.0%	17.9%	2.2%		1.4%	3.6%			
Children	66.9%	63.5%	85.8%	9.4%	0.7%		2.1%		2.7%		
post-op-2 h											
Newborns	100%	100%	85.7%	71.4%		42.9%					
Infants	82.1%	79.2%	81.1%	21.7%		3.8%	5.7%	1.9%	5.7%	6.6%	
Toddlers	71.4%	66.4%	75.0%	12.9%	1.5%	2.2%	1.5%	0.7%	3.6%		
Children	64.9%	62.8%	82.4%	5.4%	0.7%	1.4%	2.7%	2.7%	4.1%		
POD-1											
Newborns	100%	100%	100%	71.4%		14.3%					
Infants	83.9%	74.5%	87.7%	18.9%		6.6%	8.5%	1.9%	0.9%	6.6%	
Toddlers	62.8%	56.4%	73.6%	7.2%		4.3%	1.5%	0.7%	2.9%	0.7%	0.7%
Children	62.8%	59.5%	77.0%	4.1%	0.7%	1.4%	2.0%	2.7%	3.4%	0.7%	
POD-2											
Newborns	71.4%	57.1%	85.7%	42.9%		42.9%			14.3%		
Infants	48.1%	37.7%	66.9%	6.6%		3.8%	13.2%	2.9%	6.6%	3.8%	1.9%
Toddlers	22.9%	17.9%	29.3%	2.9%	1.5%	2.2%	1.5%	0.7%		1.5%	1.5%
Children	14.9%	11.5%	26.4%	1.4%		1.4%	2.1%	2.8%	2.1%	1.4%	2.1%

The 401 recruited patients included seven cases (1.7%) of newborns (age < 30 d), 106 cases (26.3%) of infants (age < 1 years), 140 cases (35.0%) of toddlers (age 1–4 years), and 148 cases (36.8%) of children (age 4–18 years).

AVP = vasopressin, Dob = dobutamine, Dop = dopamine, E = epinephrine, Intra-op = Intra-operative, Iso = isoproterenol, Mil = milrinone, NE = norepinephrine, NTG = nitroglycerin, POD = post-operative day, Post-op = post-operative, rh-BNP = recombinant human brain natriuretic peptide, SNP = sodium nitroprusside dihydrate, Trep = treprostinil.

### 3.5. Anesthesiologists and vasoactive-inotropic agents

**Figure 3.**
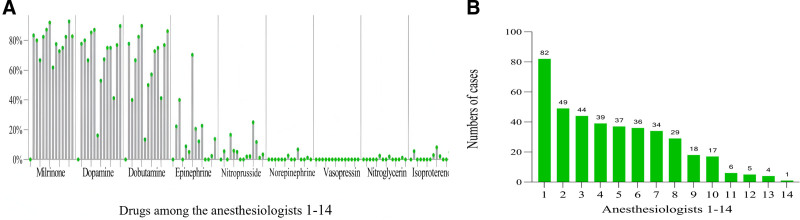
Intraoperative vasoactive-inotropic agents used by different anesthesiologists (A). In total, 14 anesthesiologists provided anesthesia care for 401 patients. Anesthesia case volume per capita ranged from 1–82 (B).

A total of 14 anesthesiologists provided anesthesia care for 401 patients, with the number of cases per anesthesiologist ranging 1 to 82 (Fig. 3B). Milrinone, dopamine, and dobutamine were the most commonly selected agents, with usage rates among different anesthesiologists ranging from 66.7% to 92.7%, 52.9% to 89.7%, and 46.2% to 86.2%, respectively. The selection of epinephrine, SNP, norepinephrine, nitroglycerin, and isoproterenol varied significantly among anesthesiologists (Fig. 3A).

## 4. Discussion

This retrospective study analyzed the use of vasoactive-inotropic agents in 401 pediatric patients undergoing cardiac surgery between April and June 2021. The key findings include: milrinone, dopamine, and dobutamine were the most commonly used inotropes both intraoperatively and postoperatively, with the combination of dopamine, dobutamine, and milrinone being the most frequent; the use of these inotropes decreased significantly by POD-2, whereas the use of vasopressin and SNP increased; and there was considerable variability in drug selection among anesthesiologists, particularly for agents such as epinephrine, SNP, norepinephrine, nitroglycerin, and isoproterenol.

Maintaining hemodynamic stability in pediatric congenital heart surgery is crucial, with vasoactive-inotropic agents playing a key role. However, their use remains debated. Some studies support their efficacy in stabilizing hemodynamics, whereas others associate them with poor outcomes, highlighting the complexity of their clinical role.^[10]^ This variability may stem from differences in practice across institutions and among ICU staff, as observed in Europe and North America.^[1,3,11]^ Determining which agent is superior for maintaining stability remains challenging, with clinical protocols often shaped by years of experience and local expertise, leading to differing practices.^[6]^

Milrinone^[12,13]^ was the most commonly used inotrope, both intraoperatively and postoperatively, consistent with previous studies. Its widespread use in pediatric cardiac surgeries is due to its ability to enhance myocardial contractility and stabilize hemodynamics, especially in patients with heart failure. Our study found a high milrinone usage across all age groups, particularly in neonates and infants, reflecting its crucial role in maintaining cardiac output in these populations. As a second-generation phosphodiesterase III inhibitor, milrinone increases intracellular cAMP, improving cardiac output and reducing pulmonary vascular resistance. However, it can cause side effects such as arrhythmias, especially in patients with preexisting cardiac conditions or those undergoing complex surgeries.^[13]^

Dopamine and dobutamine were also used frequently, though less often than milrinone, particularly postoperatively. Both agents are effective at increasing cardiac output, especially in cases of low blood pressure and heart failure. Their use declined as patients stabilized, consistent with other studies, likely reflecting improved myocardial function and reduced inotropic needs during recovery.^[11]^ Dopamine, a natural catecholamine, primarily targets α- and β-adrenergic receptors to enhance cardiac output and perfusion pressure. Despite controversy over its benefits in critically ill patients with cardiac dysfunction, a meta-analysis found insufficient evidence to support its use in adults with cardiac dysfunction. However, dopamine remains a preferred choice in lower doses due to its more pronounced inotropic effects compared with its vasopressor activity.^[14]^ Dobutamine,^[15]^ a β-adrenergic agonist, is commonly used for its positive inotropic effects but may cause dose-dependent sinus tachycardia. This side effect can also be triggered by pain, agitation, and fever, which are common postoperative issues in pediatric patients. Although arrhythmias, including sinus tachycardia, have been reported with dobutamine and milrinone, their clinical significance is unclear.^[11]^ When used appropriately, both medications are generally considered safe for pediatric cardiac surgery patients.

We observed an increase in the use of vasopressin and SNP postoperatively, especially on POD-1. Vasopressin, primarily used for refractory hypotension and vasodilation, became more prevalent as patients advanced in recovery, highlighting its role in managing postsurgical hemodynamic instability. The rise in SNP usage, particularly in neonates, suggests its importance in controlling blood pressure and systemic vascular resistance in patients with postoperative hypertension or vasodilation. Our study found that increased vasopressin use was associated with a reduced need for norepinephrine, consistent with findings by Argenziano et al.^[16]^ Additionally, Morales et al^[17]^ found that vasopressin shortened catecholamine use, reduced hypotensive episodes, and decreased ICU stay, suggesting its benefits in pediatric cardiac recovery. Meanwhile, SNP use significantly decreased by POD-2, reflecting its transient role in the immediate postoperative period before stabilization in later recovery stages.

The variability in vasoactive drug selection among anesthesiologists is notable: milrinone, dopamine, and dobutamine were commonly used, whereas drugs like epinephrine, SNP, norepinephrine, nitroglycerin, and isoproterenol showed significant differences in selection across providers. This likely reflects differences in clinical judgment, individual preferences, and patient conditions, emphasizing the need for more standardized drug protocols. Our study revealed that 14 anesthesiologists managed 401 pediatric patients, with varying inotrope usage, reflecting the individualized approach to pediatric cardiac care. Additionally, we found that inotropic support was higher in complex surgeries (category 4), aligning with previous research,^[9]^ which underscores the importance of tailoring therapy based on surgical complexity and patient risk. Moreover, younger patients, particularly newborns and infants, required more inotropic support, consistent with the notion that their underdeveloped cardiovascular systems make them more prone to instability.^[18]^ These findings highlight the importance of personalized and closely monitored inotropic therapy, especially in high-risk and younger patients.

At present, there are no robust or convincing data to support a single superior vasoactive-inotropic agent-based therapy for maintaining hemodynamic stability or improving prognosis. Postoperative vasoactive-inotropic agent therapy must be tailored to each patient and continuously adapted to specific patient needs, often involving more than one medication. Catecholamines have traditionally been used as first-line agents in many European heart centers.^[19]^ However, no large-scale studies have demonstrated a definitive outcome benefit of catecholamines compared with non-catecholamines. Therefore, we believe that the choice of drugs is still guided by their positive effects and the experience of clinicians.

This study has several limitations: As a retrospective analysis, it may introduce selection bias due to reliance on previously collected data. Drug use decisions were influenced by factors such as anesthesiologist experience and surgical complexity, which were not fully accounted for. The data were collected from a single institution (Fuwai Hospital), limiting the generalizability of our findings to other regions or patient populations. We did not assess the direct impact of specific drug combinations on patient outcomes, such as postoperative recovery or complications.

In conclusion, this study summarizes the use of vasoactive-inotropic agents in pediatric cardiac surgery at Fuwai Hospital, emphasizing the common use of milrinone, dopamine, and dobutamine. Drug selection varied by anesthesiologist experience, patient age, and surgical risk, with higher-risk surgeries showing a preference for specific drug combinations. These variations underscore the need for standardized protocols and personalized treatment strategies. By analyzing 401 patients, we establish a baseline for drug usage patterns, providing insights into current practices and guiding future research.

## Acknowledgments

Both authors are very grateful to reviews and editors for their help and suggestions.

## Author contributions

**Conceptualization:** Yan-ting Sun, Yun-tai Yao.

**Data curation:** Yan-ting Sun, Li-hong Wang.

**Formal analysis:** Yan-ting Sun.

**Methodology:** Yan-ting Sun, Yun-tai Yao.

**Software:** Yan-ting Sun.

**Supervision:** Yun-tai Yao.

**Writing – original draft:** Yan-ting Sun.

**Writing – review & editing:** Yun-tai Yao, Li-hong Wang.
